# PeanutMap: an online genome database for comparative molecular maps of peanut

**DOI:** 10.1186/1471-2105-7-375

**Published:** 2006-08-11

**Authors:** Arun M Jesubatham, Mark D Burow

**Affiliations:** 1 Department of Computer Science, Texas Tech University, Lubbock, TX 79409, USA; 2 Texas Agricultural Experiment Station, Texas A&M University, 1102 East FM 1294, Lubbock, Texas 79403, USA; 3 Department of Plant and Soil Science, Texas Tech University, Lubbock, Texas 79409, USA

## Abstract

**Background:**

Molecular maps have been developed for many species, and are of particular importance for varietal development and comparative genomics. However, despite the existence of multiple sets of linkage maps, databases of these data are lacking for many species, including peanut.

**Description:**

PeanutMap  provides a web-based interface for viewing specific linkage groups of a map set. PeanutMap can display and compare multiple maps of a set based upon marker or trait correspondences, which is particularly important as cultivated peanut is a disomic tetraploid. The database can also compare linkage groups among multiple map sets, allowing identification of corresponding linkage groups from results of different research projects. Data from the two published peanut genome map sets, and also from three maps sets of phenotypic traits are present in the database. Data from PeanutMap have been incorporated into the Legume Information System website  to allow peanut map data to be used for cross-species comparisons.

**Conclusion:**

The utility of the database is expected to increase as several SSR-based maps are being developed currently, and expanded efforts for comparative mapping of legumes are underway. Optimal use of these data will benefit from the development of tools to facilitate comparative analysis.

## Background

Molecular maps are an important part of the genomics revolution. The use of DNA markers has expanded our knowledge of genetic linkage relationships considerably by removing the need for linkage to phenotypic markers. DNA-based maps were first developed in the 1980s [1], and have since expanded to encompass thousands of markers in some species [2-4]. Two RFLP-based maps of peanut have been published [5,6], and the recent development of SSRs for peanut [7-10] is expected to result in rapid generation of additional maps.

Genetic markers have been developed for selection of qualitative traits and QTLs in multiple species. In peanut, DNA-based markers have been developed for nematode resistance, and were used for selection during of the final two generations of development of the variety 'NemaTAM' [11]. In addition to being useful in selection programs, markers are useful for identification of biological relationships among accessions and species, and are an integral part of gene isolation by positional cloning [12] and ordered gene sequencing [13].

One of the most-important insights from genomics is the discovery of synteny among species of the same botanical family and, to a lesser extent, among different families. Comparative maps using genetic markers have demonstrated considerable similarity in chromosome structure and gene order among species in the same botanical family, with Poaceae being a striking example [14,15]. Marker analysis of several legume species has also provided evidence for the conservation of gene order in Fabaceae [16]. Conservation of gene order has been used in attempts to clone genes based on comparative information between species [17], and given insights into genome structure.

One of the limitations of genomics has been the lack of informatics resources for analysis of the large amounts of data produced, and for comparison of data among species. Databases exist for species of interest as model systems or having the greatest economic value, but are lacking in other species, including peanut. Among legumes, genome databases exist only for *Glycine *[18] and *Medicago *[19]. As awareness of the significance of comparative genomics has increased, there is a trend towards databases encompassing data from multiple species. Gramene has incorporated data from maize and rice [20], and a cross-legume database, called the Legume Information System, is currently being developed to combine data from species-specific databases and permit cross-legume comparisons [21].

A large amount of the older species-specific data were held in either the AceDB-type databases [22] or proprietary databases. AceDB had the advantage that it was a database developed specifically for genomic data; however, the lack of structured query language and tools for comparative maps have made this type of database problematic for comparative genomics. Proprietary databases make use of commercial software, such as Oracle [23]; such databases are very powerful, but software licencing is prohibitively expensive for all but the largest research projects, and the software lacks built-in tools for genomics. Recently, the USDA and NIH have co-sponsored development of the GMOD [24] suite of open-source programs for genomics. This software runs on open-source databases such as MySQL [25] or PostGresSQL [26], and on multiple operating systems, including the open-source Linux operating system. Parts of the Gramene [20] and LIS [21] databases are utilizing or migrating to the GMOD-based software. The component of this software for maps is called CMAP [27].

One hindrance to the advancement of peanut genomics has been the lack of a genome database. Development of such a database would assist in the dissemination of genomic data, accelerate genomic research and varietal development, and foster comparative genomics with other legumes. In this paper, we present a new map database for peanut, called PeanutMap.

## Construction and content

The installation of PeanutMap was done on a PC with SCSI hard drives set up for Ultra 160 RAID 1 mirroring, and running the Redhat Linux Advanced Server v. 3.0 operating system [28]. The following software was installed before installing CMAP, with current versions listed after the software: libgd 2.0 [29], MySQL database v.4.0.20 [25], Perl v.5.8.0 [30], CPAN modules v. 1.390 [31], and Apache v. 2.0.46 [32]. Libgd is the C graphics library used by CMAP. The MySQL structured query language database was installed as binaries, and serves as the relational database system for data storage and retrieval. The Perl programming language is needed for execution of CMAP. The CPAN shell was installed and used to download all the required CPAN Perl modules. The CPAN shell was allowed to check automatically for dependencies among Perl modules; however, modules that the CPAN shell failed to install were installed manually. The Apache Web Server was installed as binaries, and scripts that came packaged with Apache are used to start and stop the web server.

CMAP version 0.10 was installed as the core of the PeanutMap system; CMAP is a cgi (computer gateway interface) application written entirely in Perl The CMAP software [27] is open source, originally written for the Gramene project and is now part of GMOD [24]. The locations for the cache, templates, and html documents were specified in the CMAP configuration file. Tables were then created for housing the CMAP data, using scripts that came with CMAP. A cronjob was written to remove the images from the cache folder on a daily basis.

The current data files were made in a multistage process. Linkage map data were entered in a Lotus 1-2-3 spreadsheet with columns in the order specified for CMAP. The data were exported in comma-separated variable format, and converted to tab-delimited ASCII files. The script supplied with the program was used to import the map data.

## Utility

**Figure 1:**
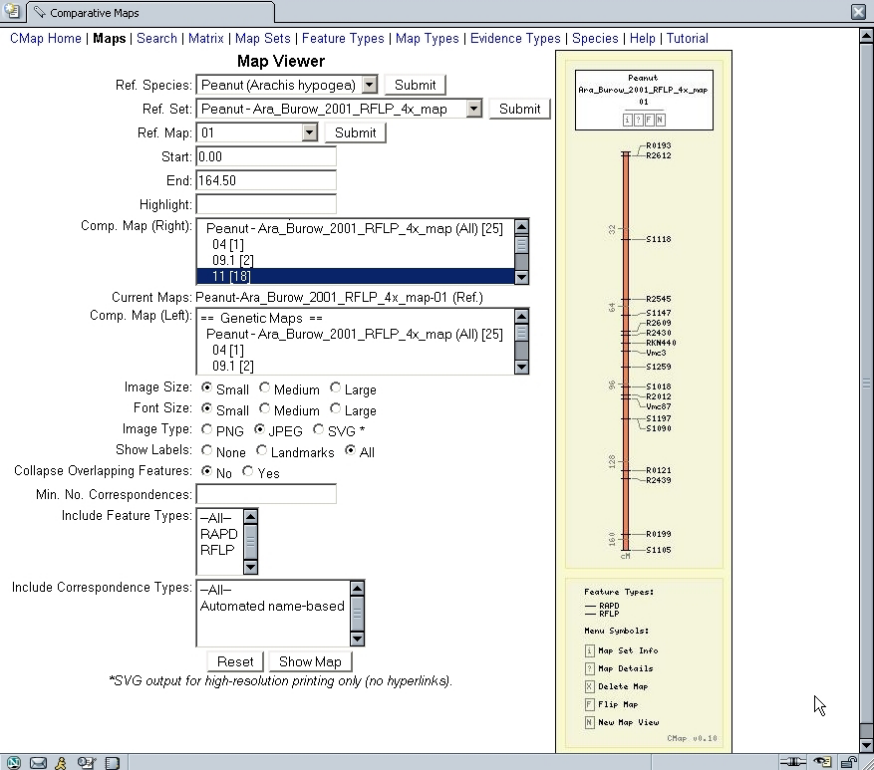
**Sample linkage group display with associated menus**. Linkage group 1 from the tetraploid map is displayed at right. At left can be seen menus for selection of map sets and maps within a set, and choices to limit the types of markers that are displayed.

The PeanutMap homepage presents 10 choices, but map display and comparison is the most-frequently used option for users. Selecting "Maps" brings up a menu that lists all the available map sets. (In the terminology of the CMAP software, a "map" is a single linkage group, and a "map set" is a collection of linkage groups, such as is found in a manuscript detailing mapping of the genome of an organism.) In PeanutMap, five map sets are available currently, two of which are large sets containing RFLP maps of the peanut genome. The first map set is from the cross *A. cardenasii *× *A. stenosperma *[5], and the other map set is from the cross between the synthetic amphidiploid TxAG-6 [*A. batizocoi *× (*A. cardenasii *× *A. diogoi*)]^4x ^and *A. hypogaea *cv. 'Florunner' [6]. The three small map sets are of markers associated with root-knot nematode resistance [33-35]. Once a particular map set is selected, a drop-down list of all linkage groups present in the set appears. When a linkage group is selected for display, that linkage map and its associated markers and map distances are drawn (Fig. 1).

**Figure 2:**
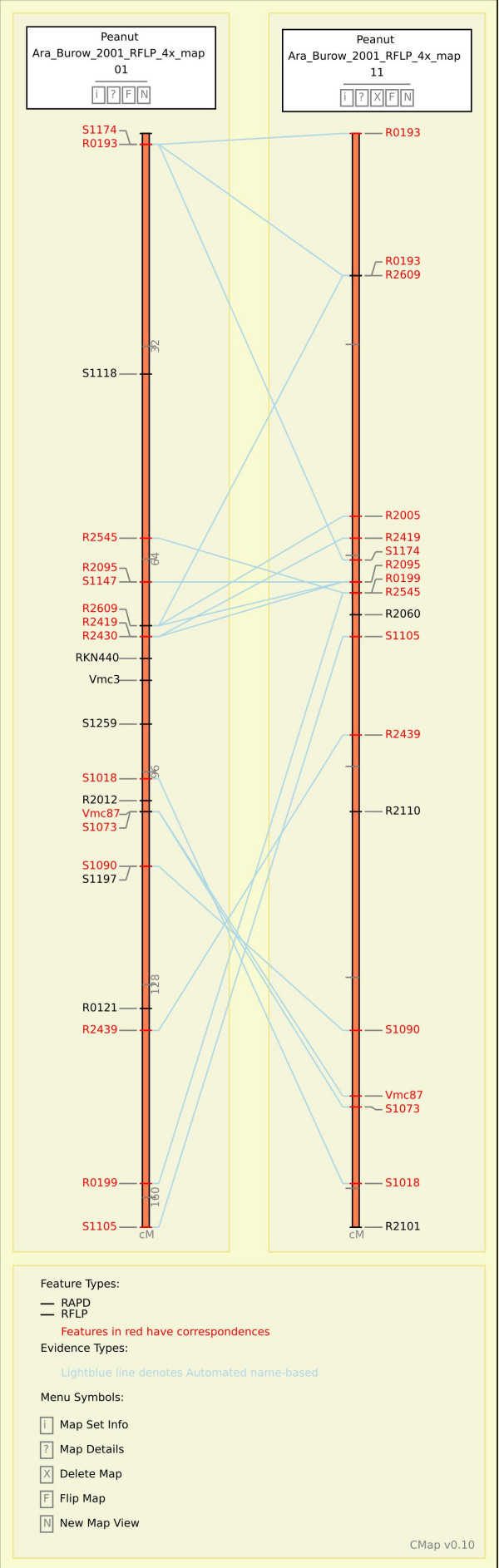
**Comparison of homoeologous linkage groups in the same map set**. Linkage groups 1 and 11 from the tetraploid map are shown; associated menus are not shown. Lines connect corresponding markers in the two maps.

The database software allows comparison among linkage groups in a map set. For example, when LG1 of the tetraploid map set [6] is displayed, the map viewer also presents a list of other linkage groups and the number of markers in common with LG1 (Fig. 1). LG11 has 18 markers in common with LG1, and selecting LG11 results in co-display of both linkage groups (Fig. 2). Markers common to both linkage groups are highlighted, and the association illustrated by lines connecting corresponding markers in the two maps.

**Figure 3:**
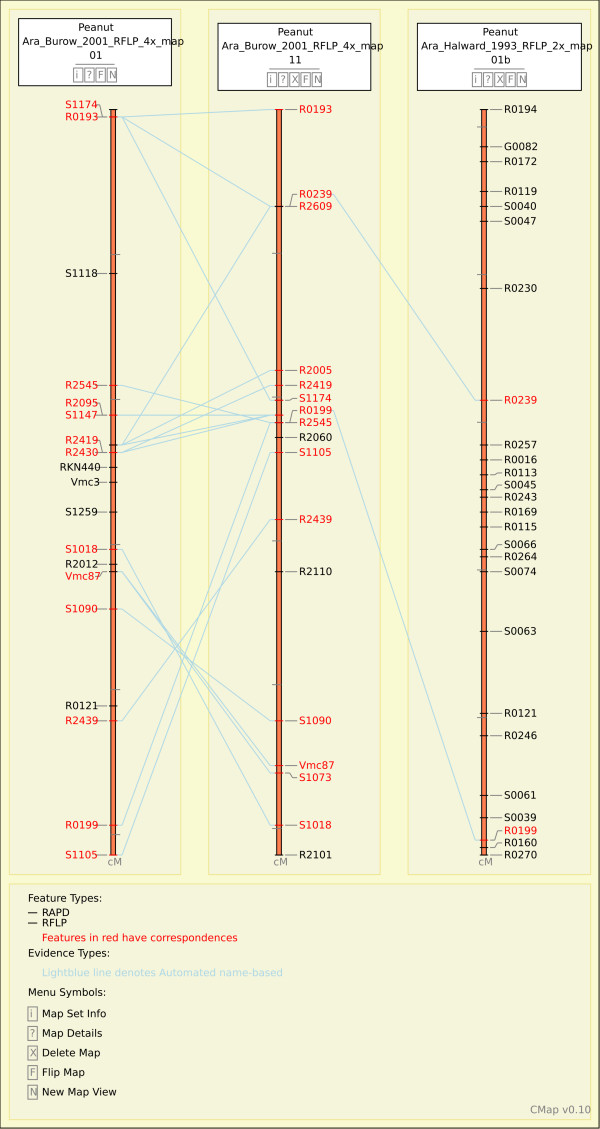
**Comparison of linkage groups within and between different map sets**. Three linkage groups are shown: linkage groups 1 and 11 from the tetraploid map, and 1b of the diploid map. Markers common to adjacent linkage groups are connected by lines.

PeanutMap can also be used to find associations among linkage groups in different map sets. For example, in the LG1–LG11 comparison above, LG1a and LG1b of the diploid map set [5] are indicated to have 2 corresponding markers (not shown); selecting LG1b brings up a figure highlighting markers common to two or more of the three linkage groups displayed (Fig. 3).

**Figure 4:**
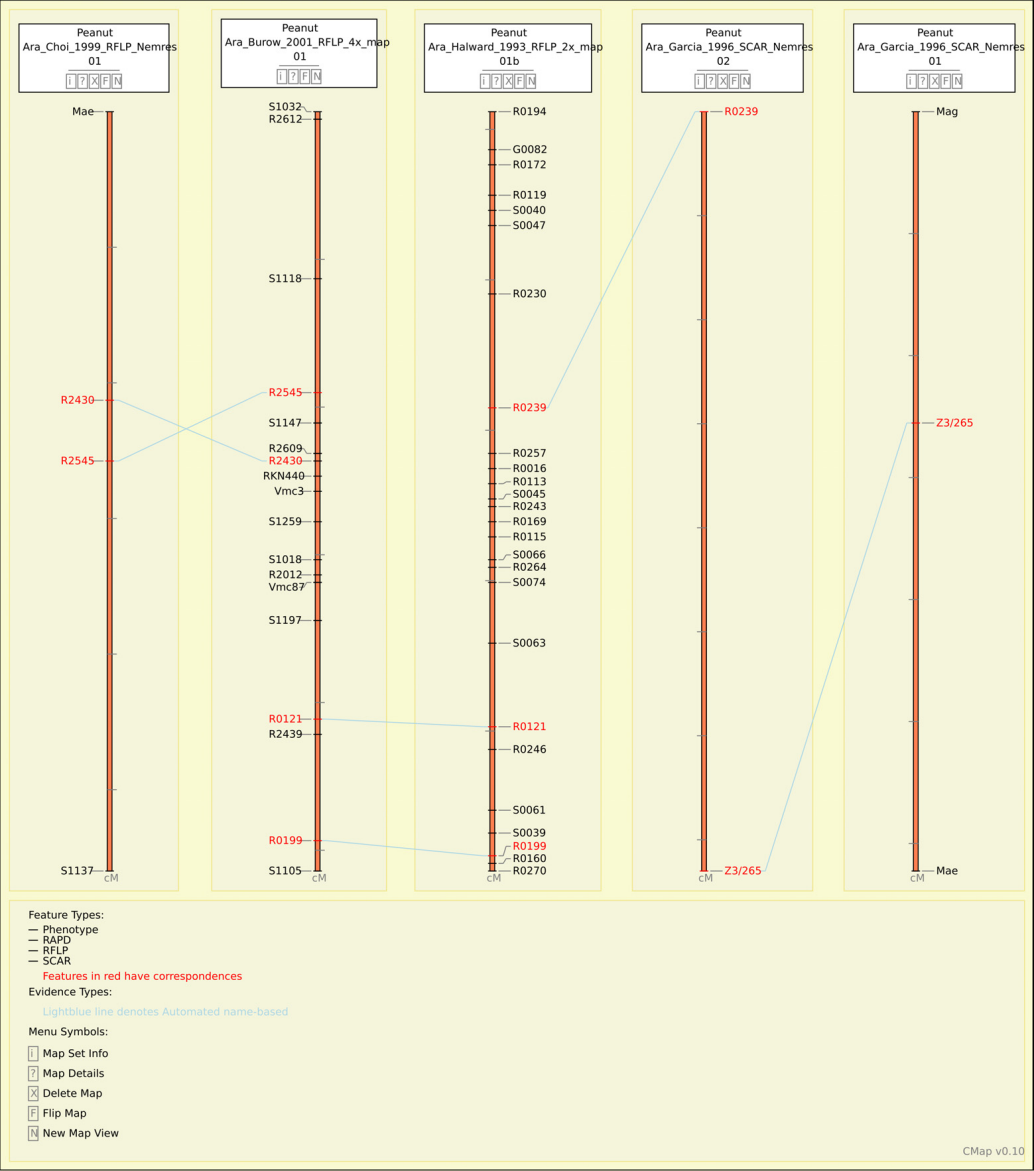
**Co-display of phenotypic and marker data**. The root-knot nematode resistance trait is denoted by 'Mae' and 'Mag', *Meloidogyne arenaria *resistance genes denoting reduced egg masses and reduced galling, respectively. Two linkage maps from genomic map sets are shown also, one of the tetraploid linkage group 1 and one of diploid linkage group 1b.

PeanutMap can also be used for display of phenotypic data. To date, only root-knot nematode resistance has been mapped, and these data are present in three small map sets. Potential relationships among these genes can be seen by displaying these map sets plus the corresponding full-length linkage groups from the large map sets (Fig. 4). In this comparison, it appears that the markers for nematode resistance [33-35] are probably located on the same linkage group, and at least some of the mapped markers could correspond to the same gene. The database is also capable of displaying mapped QTLs, and these will be added once such data become available.

The datasets in PeanutMap have been made available to the Legume Information System, which is compiling map data from different legume species [21]. This will permit data held in different legume databases to be used for comparison of synteny in gene order among different species.

## Discussion

PeanutMap is a useful addition to the tools for genetic mapping of peanut. It is the only peanut-specific genome database known to the authors. Several map sets are already present and available for use for mapping and for phenotypic analysis. It is expected that additional maps will be forthcoming, especially as SSR and SNP-based markers are mapped. We plan to update the database with additional genomic information as it becomes available.

Use of the SQL-formatted CMAP software will allow interoperability and data exchange with other genome databases, facilitating comparative mapping of peanut with other legumes and perhaps species outside the family. Incorporation of data from PeanutMap into the Legume Information System are an example of this.

## Conclusion

PeanutMap is a graphics-oriented database that makes the current peanut map data available in a web-accessible format, and allows comparative mapping of linkage data. This will undoubtedly accelerate the pace and usefulness of mapping the peanut genome, and will further allow integration of different peanut maps and facilitate comparison of peanut and other legumes.

## Availability and requirements

The PeanutMap database is web-accessible at the URL , and has been tested to work with the Netscape 7.1 [36], Mozilla Firefox 1.0 [37], and Internet Explorer 6.0 [38] web browsers.

## Abbreviations

BAC – Bacterial Artificial Chromosome

GMOD – Generic Model Organism Database

LIS – Legume Information Systems

NIH – National Institute for Health

QTL – Quantitative Trait Locus

SNP – Single Nucleotide Polymorphism

SSR- Single Sequence Repeats

RFLP – Restriction Fragment Length Polymorphism

USDA – United States Department of Agriculture

v – Version

YAC – Yeast Artificial Chromosome

## Authors' contributions

AMJ installed the software and initial version of the data, and wrote the initial draft of the manuscript. MDB conceived the idea of the database, provided direction for its installation, updated the database and web page design, and is responsible for its maintenance. He also revised and wrote the subsequent drafts of this manuscript.
